# Could Radiotherapy Effectiveness Be Enhanced by Electromagnetic Field Treatment?

**DOI:** 10.3390/ijms140714974

**Published:** 2013-07-17

**Authors:** Artacho-Cordón Francisco, Salinas-Asensio María del Mar, Calvente Irene, Ríos-Arrabal Sandra, León Josefa, Román-Marinetto Elisa, Olea Nicolás, Núñez María Isabel

**Affiliations:** 1 Department of Radiology and Physical Medicine Av. Madrid s/n, Granada 18012, Spain; E-Mails: fartacho@live.com (A.-C.F.); marsalins@hotmail.com (S.-A.M.M.); icalvente@ugr.es (C.I.); sandrariosarrabal@hotmail.com (R.-A.S.); ermtto@correo.ugr.es (R.-M.E.); nolea@ugr.es (O.N.); 2 Biohealth Research Institute of Granada C/Doctor Azpitarte 4, fourth floor, Licinio de la Fuente Building, Granada 18012, Spain; E-Mail: pepileon@ugr.es; 3 Ciber of Hepatic and Digestive Diseases CIBEREHD, C/Córcega 180 ground floor, right. Barcelona 08036, Spain; 4 Ciber of Epidemiology .and Public Health CIBERESP San Cecilio University Hospital, Avda Madrid s/n, Granada 18012, Spain; 5 Institute of Biopathology and Regenerative Medicine (IBIMER), University of Granada. Av. Conocimiento, s/n, Armilla 18100, Granada, Spain

**Keywords:** electromagnetic fields, cancer, ELF magnetic exposure, radiotherapy, genotoxicity

## Abstract

One of the main goals in radiobiology research is to enhance radiotherapy effectiveness without provoking any increase in toxicity. In this context, it has been proposed that electromagnetic fields (EMFs), known to be modulators of proliferation rate, enhancers of apoptosis and inductors of genotoxicity, might control tumor recruitment and, thus, provide therapeutic benefits. Scientific evidence shows that the effects of ionizing radiation on cellular compartments and functions are strengthened by EMF. Although little is known about the potential role of EMFs in radiotherapy (RT), the radiosensitizing effect of EMFs described in the literature could support their use to improve radiation effectiveness. Thus, we hypothesized that EMF exposure might enhance the ionizing radiation effect on tumor cells, improving the effects of RT. The aim of this paper is to review reports of the effects of EMFs in biological systems and their potential therapeutic benefits in radiotherapy.

## 1. Introduction to Electromagnetic Fields: What They Are and How They Act

Non-ionizing radiation electromagnetic fields (EMFs) is the term that broadly describes the exposures created by the vast array of wired and wireless technologies that have altered the landscape of our lives in countless beneficial ways. Although during recent years, there has been increasing public concern about potential health risks from this non-ionizing subtype of radiation exposure, many people are surprised to learn that certain kinds of EMF treatments can actually heal. Many medical appliances use EMFs in specific ways to help restore bone fractures, to heal wounds to the skin and underlying tissues, to reduce pain and swelling and for other postsurgical needs. In addition, some forms of EMF exposure are used to treat depression [1]. Most electromagnetic medical equipment uses relatively high levels of electric, magnetic or electromagnetic energy [2].

An electric field (measured in volt/meter (V/m)) exists in a region of space if a charge experiences an electrical force. A magnetic field (MF) (measured in Tesla (T) and the associated magnetic flux density exist only if electric charges are in motion, *i.e*., if there is a flow of electric current. Electric and magnetic fields are characterized by their magnitude, direction and the frequency characteristics of their sources.

At high frequencies, EMFs propagate by means of tightly coupled electric and magnetic fields (radiation). In radiofrequency fields, tissue penetration ability is frequency-dependent. In the extremely low frequency (ELF) range, electric and magnetic fields are effectively uncoupled and can be evaluated separately as if they arose from independent sources. In this situation, the electric field barely penetrates the body, and the biological effects from ELF fields are due to the action of the unshielded magnetic field.

Depending on their energy, EMFs constitute ionizing radiation when they exert ionizing properties, *i.e*., when their energy is sufficiently strong to detach electrons from their orbits around atoms and to ionize the atoms as do X-rays or γ-rays. Therefore, long-term exposure to this subtype of radiation results in harmful health effects. However, when their energy is not strong enough to ionize molecules, they are termed non-ionizing EMFs and are considered, at least conceptually, as not harmful. In general, in scientific literature, the term, EMFs, is employed only with respect to the non-ionizing

EMFs located downstream from visible light in the spectrum, and therefore, we will focus on these and use this term (EMFs) to avoid possible confusion. EMFs are traditionally classified according to their oscillatory frequency, as follows: Extremely low frequency (ELF-EMF), low frequency (LF-EMF) and intermediate frequency (IF-EMF), from 0 to 300 kHz, radiofrequency (RF-EMF, 300 kHz–300 MHz) and microwave and radar (MW-EMF, 300 MHz–300 GHz). Tumor-treating electric field (TTField) is a recent term used to describe the alternating electric fields of low intensity (1–3 V/cm) and intermediate frequency (100–300 kHz) that are generated by special insulated electrodes applied to the skin surface.

Biological systems contain EMFs that extend beyond the body. These body-produced EMFs must be distinguished from those produced outside it (*i.e*., exogenous EMFs). The former are crucial for biological systems, and alterations to them may produce physical and/or behavioral disorders [3,4]. Exogenous EMFs can be divided into two main groups: (1) natural EMFs, including the Earth’s geomagnetic field; and (2) artificial EMFs, including transformers, transmission lines, domestic electric machines, radio transmitters, *etc*. Natural fields are static or very slowly varying. The electric field in the air above the earth’s surface is typically 100 V/m, but during strong electric storms, it may increase 10-fold or more. The geomagnetic field is typically 50 μT. Obviously, natural EMFs cannot be managed, but artificial ones may be modulated if necessary, for example, when they are associated with risks to human health. Most man-made sources are at extremely low frequencies. The generation, transmission, distribution and use of electricity at 50 or 60 Hz results in the widespread exposure of humans to ELF fields on the order of 10–100 V/m and 0.1–1 μT and, occasionally, to much stronger fields. The term “electronic pollution” has been coined to reflect the fact that exogenous EMFs are probably related to health risk factors [2]. This review addresses EMFs with frequencies ranging from 0.16 Hz to 900 MHz. Although there is some controversy, the majority of data obtained from the studies summarized in this work suggest that the effects of ionizing radiation on cells and tissues are strengthened by EMF, thus supporting its use to improve RT outcome.

Because ELF fields can interact with biological systems, the interest in and concern about potential hazards are understandable. To understand the effects of electric and magnetic fields on humans, their electrical properties, as well as their size and shape have to be considered with respect to the wavelength of the external field. The main difficulty in explaining the effect of electromagnetic radiation on human health resides in our lack of knowledge about the physical-chemical mechanisms underlying life, mainly due to the enormous intrinsic complexity of biological systems. In this respect, Frölich was a pioneer, postulating several years ago that EMFs might exert biological effects, such as coherent electrical polar oscillations, the generation of EMFs in living cells [5–8] and associated disturbances in cancer cells [9]. Since then, many cases of bioelectricity in human beings have been described [2].

## 2. Biological Effects of EMFs

**Figure 1:**
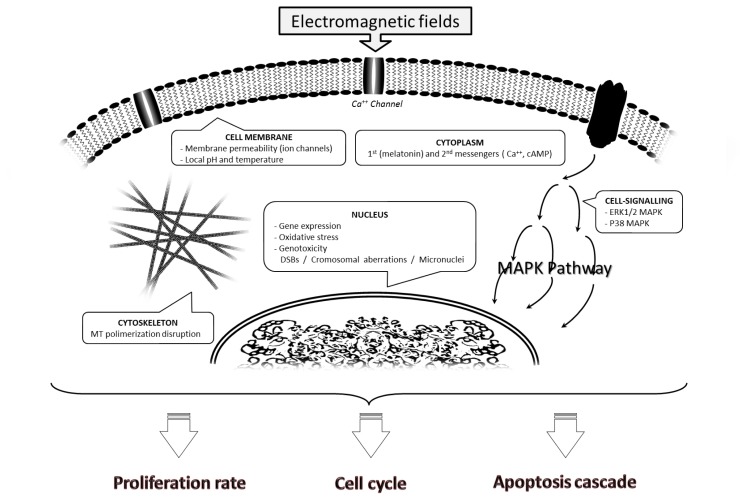
Main effects on the cell after exposure to electromagnetic fields (EMFs). EMFs alter the membrane structure and permeability of small molecules, such as Ca^2+^, causing changes in the local pH and/or temperature, and can also reorganize cytoskeleton components. It has been suggested that microtubule polymerization may be disrupted by external intermediate-frequency EMFs (100–300 kHz). The EMF-derived alteration of cell-signaling pathways (extracellular-regulated kinase (ERK)1/2 mitogen-activated protein kinase (MAPK) and P38 MAPK) would be provoked by the dysregulation of ions or by alterations in melatonin and second messengers, such as Ca^2+^ or AMPc. EMF exposure also causes gene expression modifications and DNA-related damage involving free radical production, affecting DNA structure and provoking strand breaks and other chromosomal aberrations, such as micronucleus formation. All these changes ultimately influence cell cycle progression and the rate of proliferation and apoptosis.

The enormous complexity of the molecular mechanisms underlying cell responses to EMF exposure and the lack of consensus regarding treatment schedules are at the heart of the present controversy regarding the biological effects of EMFs (Figure 1). Furthermore, a wide variety of EMFs may be involved, and their consequences may differ widely. In the following paragraphs, we discuss some research findings and attempt to clarify what might take place within the cell after EMF exposure, distinguishing among different EMF treatments (Table 1 summarizes *in vitro* findings reviewed in this paper and the main characteristic of an applied field).

### 2.1. Effects on the Cell Membrane

Although there is still no consensus among biophysicians as to whether EMFs traverse the cell membrane, it is generally thought that EMFs have great difficulty in passing through the plasma membrane [10]. Nevertheless, membrane permeability can be transiently increased by applying an electric field, and this impulse might be chemically transmitted. This process has been described in several papers, and many different theories have been postulated about the underlying physical phenomena. Electrical stimulation is known to cause changes in the local pH and/or temperature [10], and this could lead to membrane destabilization. Intramembrane proteins located in the lipid bilayer, which function as ion channels, may also significantly contribute to this, since EMF exposure leads to the formation of ion channels without cell control, thus inducing severe metabolic modifications [10]. Theoretical models have corroborated this observation, suggesting that a coherent modulation of ion channel gating by an external signal leads to the occurrence of a periodic component in the ion current across a membrane containing a system of multiple channels [11]. This has also been theoretically demonstrated for MW-EMF exposure [12]. We now conduct a brief review of the literature concerning the intracellular alterations observed, preceded or otherwise by membrane alterations, after EMF exposure.

### 2.2. Effects on the Cell Cytoskeleton

Cell cytoskeleton physiology is responsible for crucial events within the cell, with intracellular transport and cellular division being the most significant of these. In the literature, there is some controversy concerning the influence of EMFs in cytoskeleton physiology. Although some researchers have reported there is no influence of EMFs on the cytoskeleton and/or motor proteins in murine macrophages [32], many studies have shown that cytoskeleton components are reorganized after exposure to EMFs [13,33]. Kirson *et al*. suggested that microtubule polymerization may be disrupted by external intermediate-frequency (100–300 kHz), alternating electric fields; likely to influence chromosome alignment and separation. This finding implies that EMFs may affect the generation of mitosis aberrations, leading to mitotic arrest and a decreased rate of proliferation [14].

### 2.3. Effects on Cytoplasm

The biological implications of membrane interactions between EMFs and cells are believed to affect the cytoplasm through different cell-signaling pathways. In most cases, the EMF-derived alteration of these pathways would be provoked by the dysregulation of ion channels (as explained above) or by alterations in hormones and second messengers, such as Ca^2+^ or cyclic adenosine monophosphate (cAMP), although other messengers are also altered by exposure to EMFs [34].

#### 2.3.1. Disruption of First and Second Messengers

Exactly how MFs disrupt cancer promotion has yet to be established, but the disruption of circulating levels of melatonin has been proposed as a plausible factor in this respect [15,35,36], and recent data indicate that MF exposure also impairs the effects of melatonin within the cell. Melatonin is a lipophilic hormone that can directly cross the membrane and bind to different receptors. In all cases, the melatonin membrane receptors are coupled to second messenger cascades, which vary in cell, tissue and species-specific ways [37].Thus, many cell features, including cell proliferation, apoptosis and cell cycle progression, are affected by melatonin levels [38]. MF exposure has been reported to impair the effects of melatonin at the cellular level [35,39], although this effect appears to vary according to different experimental conditions.

EMFs can influence the transport of Ca^2+^ and, hence, its homeostasis [16,17,40]. The processes involved may include the ATPase-dependent Ca^2+^ pumps present in the endoplasmic reticulum and in the plasma membrane, the Na^+^/Ca^2+^electroneutral exchanger and the VOC (voltage-operated calcium channels)-dependent Ca^2+^ channels [40]. Ca^2+^ levels may play an important role in modulating cell proliferation, differentiation, apoptosis and cytotoxicity, as well as contributing to the action mechanisms of anticancer agents [41].

cAMP, a second messenger used for intracellular signal transduction and which conveys the cAMP-dependent pathway, leads a phosphorylation cascade that can activate different pathways. Many researchers have suggested that a deregulation of the cAMP pathways and an aberrant activation of cAMP-controlled genes is linked to apoptosis induction and tumor development inhibition in some cancers [42–44]. ELF-MF treatment has been shown to increase cAMP in a variety of *in vitro* and *in vivo* models, including monolayers, spheroids, mice and rats [17,45,46].

#### 2.3.2. Alterations in the MAPK Pathway

It has been suggested that exposure to ELFs-EMFs may affect various cell signaling pathways, including extracellular-regulated kinase (ERK), the 1/2 mitogen-activated protein kinase (MAPK) pathway [18,19,47] and the P38 MAPK pathway [20,47].

MAPKs are a broad family of protein serine/threonine kinases, divided into three major subfamilies. When phosphorylated, the signal is translocated to the nucleus, and gene transcription is activated. We focused on the MAPK pathway, since it is considered the most important one involved in the DNA damage response after ionizing radiation, with the ERK1/2 MAPK subtype being the best-known pathway affected by EMF exposure [18]. Because it is usually modulated by growth factors, but also by cAMP and Ca^++^ second messengers [21,22], ERK1/2 has been linked to cell proliferation, senescence, differentiation and apoptosis, whereas stress-activated protein kinase/C-Jun *N*-terminal kinases (SAPK/JNK), another MAPK subtype, has been associated with cell response to inflammation and stress conditions [48]. Jin *et al*. showed that EMFs are capable of inducing ERK1/2 phosphorylation, whereas the SAPK/JNK pathway is not induced in leukemia and breast cells [18]. Other authors have reported similar results in the same cell lines concerning ERK1/2 and SAPK/JNK activation [47,49]. However, using RF-EMF, both SAPK/JNK and ERK1/2 MAPK subtypes are increased in human neuroblastoma cells [19]. With respect to mobile phone frequencies, Friedman *et al*. hypothesized that ERK1/2 may be activated by matrix metalloproteinase, released by the EGF (epidermal growth factor), resulting from the generation of reactive oxygen species [47].

### 2.4. Nuclear Effects

#### 2.4.1. Gene Expression

Gene expression is meticulously regulated by many pathways. AP-1, a heterodimeric transcription factor composed of proteins belonging to the c-Fos, c-Jun, ATF and JDP families, regulates gene expression in response to a variety of stimuli, controlling a number of cellular processes, including differentiation, proliferation and apoptosis [18]. MFs induce an increased binding activation of AP-1 [50]. Moreover, Jin *et al*. have demonstrated that AP-1 is induced by EMFs [18].

RNA binding proteins (RBPs) are another category of cellular regulators of gene expression at the transcriptional level. RBPs usually consist of two subunits, the RNA binding subunit (RBS) and the RNA binding regulatory subunit (RS). Han *et al*. pointed out that MFs increase RS levels and suggested that RS may also participate in cellular response to MF treatment in the MCF-7 breast cancer cell line [51].

Furthermore, the activation of the Egr-1 gene may be affected by RF-EMFs together with a significant decrease in mRNA levels of both Bcl-2 and survivin genes [19].

#### 2.4.2. DNA Damage

Although it is generally accepted that 50/60Hz frequency EMFs might not transfer enough energy to cells to damage DNA directly, certain cellular processes, such as free radicals, may be altered by exposure to EMF, indirectly affecting DNA structure and provoking strand breaks and other chromosomal aberrations, such as micronucleus formation or effects on DNA repair [23].

##### 2.4.2.1. Oxidative Stress

Oxidative stress, originated by a misbalance between antioxidant molecules (e.g., glutathione, GSH) and reactive oxygen and nitrogen species (ROS and RNS) is crucially involved in carcinogenesis promotion [24,52], due to the resulting genomic instability [53]. Consequently, *in vitro* studies assessing the role of ROS in biological effects triggered by EMF have been included as a high-priority research line in the World Health Organization Research Agenda (2007).

Rats treated with chronic exposure to RF-EMF have presented decreased antioxidant enzymes, such as superoxide dismutase (SOD) or glutathione peroxidase (GSH-PX), and reduced cellular antioxidant potential [54]. Similar results have been found in rat lymphocytes treated with iron ions [55] and in rabbits [56]. In addition, a recent paper showed that healthy rats given extremely low frequency EMF treatment also presented increased ROS production, with higher levels of thiobarbituric acid reactive substances (TBARS) and hydrogen peroxide (H_2_O_2_) in heart samples after 40 Hz, 7mT, 60 min/day EMF exposure for 14 days. The antioxidant capacity in plasma was also decreased. However, when this treatment schedule was reduced to 30 min/day, no significant decrease in redox status was detected [27]. With respect to cancer research, a number of papers have been published assessing changes in redox status, including neuroblastoma cells [28], leukemia cells [57] and fibroblasts [25,26]. In THP-1 acute leukemic cells, EMF reduces antioxidant enzyme activity and enhances nitrogen intermediates involving the iNOS pathway [57]. According to Friedman *et al*., when HeLa cells are treated with short exposures to RF-EMF, there is an increase in the activity of NADH oxidase, a membrane enzyme, thus inducing ROS production. In consequence, matrix metallo proteinases (MMPs) are synthetized, and these, in turn, release the epidermal growth factor and activate the ERK pathway [47].

##### 2.4.2.2. Double Strand Breaks, Chromosomal Aberrations and Micronucleus Induction

A survey of the literature suggests that only intermittent EMFs might exert genotoxic cell effects and that cells exposed to continuous EMFs suffer no DNA damage [28,58,59]. In their study of both continuous and intermittent EMF exposures, Ivancstis *et al*. observed genotoxic effects only when different schedules of intermittent EMFs were used. These authors suggested that subjecting cells to a constant field may induce adaptive mechanisms, protecting the genome from harmful influences, whereas regular changes in environmental conditions might interfere with these mechanisms and lead to DNA impairment in human fibroblasts [60]. Focke *et al*. extended this study to different human cell models [29]. Kim *et al*. reported increased levels of γ-H2AX in HeLa and normal lung fibroblasts after daily MF exposure [20]. DNA damage, such as double strand breaks (DSBs) or DNA impairment, leads to genomic instability. In this respect, Winker *et al*. found that the exposure of cultured human fibroblasts to 50 Hz, 1mT, 15h EMFs resulted in genomic instability marked by increases in chromosomal aberrations [59]. These authors also reported the induction of micronucleus formation after EMF exposure [59]. Micronuclei are cytoplasmic bodies in which part of the acentric chromosome or the whole chromosome is not carried to the opposite pole during anaphase. Their formation has traditionally been associated with genotoxic activity, and micronucleus tests are widely used to evaluate potential carcinogens, since this test has been described as a good biomarker for DNA damage. Micronucleus aberrations could also lead to apoptosis [59].

### 2.5. Modulation of Proliferation Rate and Cell Cycle Progression

Cell viability is necessary for a proper response to endogenous factors, such as soluble hormones, growth factors, effectors and genes, and also to exogenous factors, such as cytotoxic drugs and environmental stress [30]. It was recently suggested that repetitive ELF magnetic field exposure with 6 mT decreases cell viability by inducing DNA double strand breaks [20].

Cell proliferation is a complex process controlled by multiple cell signal transduction pathways. Controversial data have been published concerning the enhancement [31,61], inhibition [62,63] or non-effect of MF exposure with respect to cell proliferation [64,65]. Nevertheless, the scientific contribution of Pasche’s lab seems to be clarifying the potential role of EMFs in proliferation rate control. These authors developed a novel approach to treat advanced hepatocellular carcinoma (HCC), consisting of the intrabuccal administration of very low levels of RF-EMF, amplitude-modulated at specific frequencies in patients with cancer [66]. In a single-group, open-label, phase I/II study consisting of 41 patients with advanced HCC and limited therapeutic options, they observed that treatment with intrabuccally-administered amplitude-modulated electromagnetic fields is safe, well tolerated and shows evidence of antitumor effects in patients with advanced HCC [67]. In a recent *in vitro* study, the same authors demonstrated that cell proliferation rates in hepatocellular carcinoma and breast cancer cell lines decrease after exposure to EMFs [68]. Focke *et al*., using fibroblasts [29], and Kirson *et al*., using glioma and melanoma cells [69], also observed a significant inhibition of cell proliferation, suggesting that the optimal frequencies might differ among cancer cell types, being inversely related to cell size [14,69]. Kirson *et al*. carried out *in vivo* experiments and found that tumor growth was also decreased in mouse models for both breast and non-small-cell lung carcinomas after exposure to alternating electric fields [14].

Few studies have been undertaken to assess the role of EMFs in cell cycle progression. Some papers have suggested that there are cell cycle delays in different steps. Kim *et al*. have reported increased levels of phosphorylated checkpoint kinase 2 (ChK2) after 60Hz ELF-MFs in HeLa and lung fibroblasts [20], a typical cell cycle checkpoint induced after ionizing radiation-induced DNA damage through the ATM/ATR pathway. Lange *et al*. found that MFs exerted an inhibitory effect on the G1-phase promotion induced by the altered expression of p16INK4a and p21CIP1. These two proteins are involved in G1 arrest through the destabilization of p53 and the inhibition of cyclinD/Cdk complexes [70]. Since EMF could alter both microtubule polymerization and RBS proteins, thus provoking replication disturbances, it seems reasonable to suggest that EMF exposure may accumulate more cells in the S-phase than in the other cell cycle phases. EMF-exposure causes a significant increase in the percentage of Rat-1 fibroblasts in the S-phase at 12 and 48h, whereas a 30% S-phase decrease has been observed 72 h after EMF exposure, compared to controls [31]. Interestingly, the latter authors also found a significant increase in both DNA strand breaks and 8-OHdG levels, which reached maximum values after the S-phase peaks [31]. RF-EMF treatment has been found to arrest cells in the G2/M phase in human neuroblastoma cells [19]. Using RF-EMF and confocal laser scanning microscopy, Zimmerman *et al*. found over 60% mitotic spindle disruption in HepG2 cells receiving hepatocellular carcinoma (HCC)-specific RF-EMF, compared with cells not receiving exposure [68]. Nevertheless, Ruiz-Gómez *et al*. have reported similar cell cycle patterns between unexposed and 25Hz frequency EMF-exposed leukemia and colon carcinoma human cell lines [71].

### 2.6. Induced Apoptosis Cascade

MFs affect several important physiological processes related to the mitochondrial membrane potential, such as ATP synthesis, Ca^2+^ flux and cytochrome c release to the cytosol [72]. Thus, cytochrome c binds to apoptotic protease activating factor-1 (Apaf-1) and ATP to create the apoptosome, capable of triggering apoptosis induction via caspase-9 cleavage [73]. Chromosome and mitotic spindle aberrations also contribute to apoptosis initiation. Therefore, changes in apoptotic rate after EMF exposure could reasonably be expected. Focke *et al*. reported a slight increase in the percentage of apoptotic fibroblasts after exposure to intermittent EMFs [29]. ELF-EMF also induces apoptosis in tumor cell lines [62]. In most cell types, RF-EMF increases the expression of Egr-1, a strong transcriptional activator of key genes involved in the cell death pathway [19]. Bcl-2 decreases and the apoptosis rate increases after RF-EMF-exposure in human neuroblastoma cells [19]. MW-EMF also induces time-dependent apoptosis in human epidermoid cancer cells [74].

Evidence is accumulating that components of the plasminogen activator (PA) system are somehow involved in cell death processes. It has been suggested that plasminogen may be linked to apoptosis mediated by cycloheximide (CHX) [75], dramatically increasing the rate of CHX-induced apoptosis. The latter authors suggest that PA may play a role in the degradative (*i.e*., late-stage) events of cellular apoptosis [75]. Furthermore, the plasminogen activator system could play an important role in EMF-induced apoptosis through the enhancement of the urokinase plasminogen activator (uPA) [76]. P38 has also been postulated as influential in the induction of apoptosis following EMF exposure. Thus, Kim *et al*. detected that p38 is only activated upon exposure to a 60 Hz frequency and 6mT MF for 30 min every 24 h for three days. This activation was found after 48 h and 72 h of exposure in HeLa cancer cells and IMP90 lung fibroblasts, respectively. These results suggest that repetitive exposure to a small dose of EMF may induce stress and lead to activation of p38 and other apoptosis-related signaling pathways [20].

## 3. EMFs and Magnetic Fields as Useful Adjuvants during Radiotherapy

EMFs are clinically useful in physical medicine to stimulate bone fracture healings [77], as well as to mitigate chronic pain or edema. However, the biological effects of EMF treatment are not completely elucidated, and there remain possibilities for its inclusion in other medical areas, such as cancer treatment [78,79].

### 3.1. Genotoxic Effect of Ionizing Radiation

Ionizing electromagnetic radiation is a type of radiation that is frequently used in the treatment of patients with radiotherapy (RT). Typical energies of the photons produced by 4–25 megavolts (MV) are linear accelerators in the RT range from less than 100 kiloelectron volts (keV) to several megaelectron volts (MeV). Although ionizing radiation deposits its energy randomly, causing damage to all molecules, there are multiple copies of most molecules (e.g., mRNA, proteins), which undergo continuous, rapid turnover, thus limiting the consequences of this damage. In contrast, DNA is present in only two copies and has very limited turnover. It is the largest molecule, provides the largest target and is central to all cellular functions. Ionizing radiation can affect DNA directly, generating charged particles or electrons with the kinetic energy of photons (X and γ rays), breaking phosphodiester linkages. This represents around 30% of all DNA damage and is termed the direct action of radiation [80,81]. Other radiation injury is derived from ionizing radiation-raised free radical action and is known as the indirect action of radiation. Briefly, the hydroxyl radical, of considerable biological significance, is formed when ionizing radiation interacts with water molecules, in a process called “water radiolysis”. As a result, free radicals are produced and DNA is harmed. The successful use of radiation to treat cancer results primarily from its ability to cause the death of individual tumor cells, mainly influenced by the amount of DNA damage accumulated, which implies that any agent that increases DNA damage or/and stimulates cell death might potentially improve RT effectiveness.

### 3.2. *In Vitro* Effects of EMF and Ionizing Radiation

Although ELF-EMF does not transmit enough energy to affect chemical bonds, it may mediate cell death as a result of the DNA damage induced by oxidative stress, acting as a carcinogen by inducing DNA instability. However, to date, few studies have been performed to assess the potential role of EMFs in RT. In 1999, Miyakoshi *et al*. suggested that MFs enhance the X-ray mutation rate in ovarian cells [82]. Ding *et al*. reported similar results in a glioma-derived cell line [83]. Both results might be correlated with DNA instability and, thus, with radiosensitivity. Using a very sensitive method to detect DNA damage involving microsatellite sequences, it has been confirmed that not only is ELF-EMF mutagenic as a single agent, but also that it can potentiate the mutagenicity of ionizing radiation exposure in glioma cells [84].

Abnormalities in mitotic spindle formation have traditionally been associated with increased apoptosis rates and, thus, with cell radiosensitivity [85]. Therefore, microtubule polymerization disrupted by EMFs may significantly increase apoptosis rates triggered by X-rays. Because it is generally considered that micronuclei containing kinetochore proteins are formed by the lagging of whole chromosomes, the frequency of kinetochore-positive micronuclei can be correlated with genomic instability [86]. The induction of lagging chromosomes has generally been attributed to a dysfunction in the spindle apparatus, such as disruption of the microtubules [86]. Ding *et al*. reported increased kinetochore-positive micronuclei formation in irradiated cells previously exposed to ELF-MFs compared with non-exposed irradiated cells [86]; similar results have been reported by Lagroye *et al*., using EMFs and gamma radiation [87].

Synergistic effects seem to be the most plausible explanation of the potential role of EMFs during RT. Several studies have investigated the ability of ELFs-MFs to enhance ionizing radiation-induced cytotoxicity and genotoxicity [88]. The existence of cooperative effects between EMFs and X-rays in apoptotic rate induction in a liver cancer cell line has also been demonstrated [89]. An EMF-enhanced key activator of apoptosis cascade, Egr-1, has been postulated as a promising orchestrator between EMFs and ionizing radiation during apoptotic rate induction [19,90,91]. Taking into account that the promoter of Egr-1 contains radiation inducible CArG DNA sequences, consistent results have been developed [90]. Moreover, a radiosensitizer role has been attributed to Egr-1 in melanoma cells [92], and thus, EMFs could exert a synergistic role in radiosensitivity response. Induced P38 MAPK has also been revealed by repeated exposure to a time-varying magnetic field leading to apoptosis cascade promotion in normal and cancer human cells [20]. Enhanced P38 MAPK activity after ionizing radiation exposure has been reported by many research groups [93–96]. De la Cruz-Morcillo *et al*. recently reported that ionizing radiation activates p38 MAPK in a p53-dependent fashion, and that activation of the MKK6/3-p38MAPK-p53 signaling axis leads to apoptosis [94]. In addition, an important degree of involvement of p38 MAPK in cell radiosensitivity has been suggested [97].

Radiosensitivity also depends on the cycle phase of cells during ionizing radiation exposure. It has been shown that cells irradiated during the G2/M-phase are more radiosensitive than when this takes place in other cell cycle phases [80]. Therefore, EMFs causing arrest of the cells in the G2/M-phase might also be useful in radiosensitizing tumor cells [19,31]. P38 MAPK activation has also been related with radiation-induced G2/M arrest [98]. All these results are indicative of synergistic effects by non-ionizing radiation-EMF and ionizing radiation-EMF on the cellular radiosensitive response through p38 activation.

### 3.3. *In Vivo* Effects of EMF and Ionizing Radiation

Conceptually, local tumor control is another interesting endpoint for both clinical and experimental research into improving RT effectiveness. A tumor is locally controlled when all of its clonogenic cells (*i.e*., cells with the capacity to proliferate and to cause recurrence after radiotherapy) have been inactivated [85]. The therapeutic benefit would require a differential effect between tumor cells and normal tissue in order to allow for enhanced radiotherapy effectiveness without increased toxicity. In this sense, Wolf *et al*. [31] have demonstrated differences in DNA damage after exposure to ELF-EMFs in both normal and tumor cells. The higher level of DNA damage found in neoplastic cells was associated with defects in repair mechanisms, a common feature of tumor cells. These authors also found that antioxidant treatment (α-tocopherol at 10 μM) prevented stimulation of cell proliferation in normal cells, but not in tumor cells. Differences in DNA damage were also described for normal and tumor cells after this antioxidant treatment. These results show a differential EMF effect between tumor and normal cells. Taking into account the impaired repair capacity of tumor cells, this differential effect could be extended to ionizing radiation treatment, thus supporting the view that the outcome for cells exposed to ionizing radiation could be strengthened by EMF. Experiments carried out on hepatoma-implanted mice have shown that five periods of combined 100 Hz frequency MFs and 4 Gy X-ray could significantly extend the overall days of survival and reduce the tumor size, compared to MF or X-ray treatment alone [79], suggesting that 100 Hz frequency MF could synergize with X-ray treatment in terms of survival improvement and tumor inhibition in hepatoma-implanted mice. In another study, the combination of EMF and γ-ray exposure produced a synergistic effect by triggering stress response, which increased reactive oxygen species [99].Cameron *et al*. reported a synergistic reduction of the growth rate of breast cancer xenografts in EMF/ionizing radiation mice, compared with ionizing radiation therapy [78]. They also reported that the continued daily use of EMFs following a course of ionizing radiation therapy suppressed tumor blood vessel volume density, compared to mice given the ionizing radiation therapy alone. Although ionizing radiation therapy initially interferes with tumor vascularization, leading to tumor hypoxia, which, in turn, enhances the radio-resistance capacity of tumor cells, these hypoxic areas produce HIF-1, leading to the production of angiogenesis growth factors and stimulating further tumor growth. This known sequence of events following ionizing radiation treatment has led researchers to consider ionizing radiation treatment schedules coupled with the use of an anti-angiogenesis agent. Cameron *et al*. showed that continued daily EMF therapy applied to ionizing radiation-treated mice suppressed the return of blood vessel volume density within the tumor, retarding its vascularization, growth and metastasis [78].

## 4. Conclusions and Future Directions

During the last decade, a deep sense of unrest has arisen within society concerning the possibility that human carcinogenesis might be influenced by exposure to extremely low frequency EMFs, now that this type of radiation has become part of everyday life (in diverse areas, from telecommunications to domestic utensils). Recent epidemiological studies have provided evidence of a correlation between chronic exposure to EMFs and an increased incidence of brain, breast and hematological malignancies [100–103]. However, these results are not universally accepted, and the International Agency for Research on Cancer has classified EMF as a 2B carcinogen, taking the view that the epidemiological evidence is “limited” for childhood leukemia and “inadequate” for all other cancers [2]. This classification is also supported by limited evidence in humans and inadequate evidence from experimental animal studies.

In this review, we show that several *in vitro* studies have explored the potential impact of EMF and ELF magnetic field effects on the membrane structure and permeability of small molecules, such as Ca^2+^, and on cell proliferation [68], apoptosis [62], genotoxicity [20] and cytoskeleton status [14]. Moreover, these agents have been shown to interfere with chemical reactions involving free radical production, interacting with DNA and hydroxyl radicals, which, in turn, might result in single and double strand breaks [20,60]. Taken together, the data collected in this review seem to indicate that an adjuvant EMF and/or ELFMF treatment enhancing DNA damage may be a plausible means of increasing the ionizing radiation-derived effects on cell and tissues and, thus, increasing RT effectiveness.

Although these studies reflect encouraging results and corroborate the hypothesis that combined exposure to the above agents and ionizing radiation should be used to increase DNA damage, further studies, both *in vitro* and *in vivo*, should be conducted to validate the effectiveness and therapeutic benefits of this use of radiosensitizing agents in cancer RT. Moreover, the findings presented in this paper imply that the cellular response to EMF and/or ELF MFs is highly variable among cell lines and/or specimens, affecting certain cell types, in particular. Thus, further evaluation is required to determine their possible clinical relevance, especially in combination with RT.

## Figures and Tables

**Table 1:** Main characteristics of field applied and findings from *in vitro* studies.

Cell compartment	Frequency	Intensity	Time	EMF type	Biological effect	Reference
**Cell membrane**	25 pulses/s	Peak: 0.25 T; Average: 0.5 T	238 μs 1000 pulses/day	AC MF	Changes in the local pH and/or temperature	[10]
	<100 Hz	<100 μT	0–8 ns	MF	Formation of ion channels	[11]
	MW (≈1 GHz)	>105 V/m	Several periods of oscillation	EF		[12]

**Cell cytoskeleton**	50 Hz	0.5–1.5 mT	45 min	MF	No influence of EMF on cytoskeleton and/or motor proteins	[13]
	50 Hz	2 mT (rms)	72 h	MF	Changes in microtubule polymerization	[14]
	100–300 kHz	2 V/cm	24 h	AC EF		[15]

**Cytoplasm**						

**1st/2nd messengers**	60 Hz	1.9–11.95 mG	–	MF	Melatonin involved in transmission of EMF into the cell	[16]
	50 Hz	Peak: 3 mT	24 h	EMF	Influence the transport of Ca^2+^ and, hence, its homeostasis	[17]
	50 Hz	2 mT	5 min	MF	Deregulation of the cAMP concentration	[18]

**MAPK pathway**	60Hz	0.8–300 μT	30 min	EMF	Induction of ERK1/2 phosphorylation	[19]
	875 MHz	0.005, 0.03 and 0.11 mW/cm^2^	30 min	EMF (S)		[20]
	900 MHz	1 W/kg	24 h	EMF (SAR)		[21]
	875 MHz	0.10 mW/cm^2^	30 min	EMF (S)	P38 MAPK activation	[20]
	60 Hz	6 mT	30 min/day;3 days	AC MF		[22]

**Nucleus**						

**Gene expression**	60 Hz	0.8–300 μT	60 min	MF	Increase of AP-1 transcription factor	[19]
	60 Hz	8 μT	20 min field-on; 20 min field-off			[23]
	50 Hz	0.4 mT	20 min	MF	Increase RNA binding protein levels	[24]
	900 MHz	1 W/kg	–	EMF (SAR)	Increase in mRNA levels of Egr-1, Bcl-2 and survivin genes	[21]

**DNA damage**						

***1. Oxidative stress***						

**1.1. Antioxidant system**	50 Hz	1 mT rms	24 h	AC MF	↓ SOD activity	[25]
	50 Hz	1 mT	<96 h	MF	= SOD activity	[26]
	50 Hz	1 mT rms	24 h	AC MF	↓ Catalase activity	[25]
	50 Hz	1 mT	<96h	MF	Glutathione *S*-transferase	[26]
	50 Hz	1 mT	<96h	MF	Glutathione peroxidase	[26]
	50 Hz	1 mT	<96h	MF	↑ Reduced/total GSH ratio	[26]

**1.2. Pro-oxidant molecules**	50 Hz	1 mT rms	24 h	MF	↑ iNOS	[25]
	930 MHz	5 W/m^2^	5–15 min	CW EMF (S)	↑ ROS	[27]
	900 MHz	2 W peak 0.02 mW/cm^2^	30 min/day; 7 days	EMF (S)		[28]
	50 Hz	1 mT rms	24 h	AC MF		[25]
	50 Hz	1 mT	<96 h	MF	= ROS	[26]

***2. Genotoxicity***	60 Hz	6 mT	30 min/day; 1–3 days	AC MF	Increased levels of γH2AX	[22]
	50 Hz	1 mT	15 h, 5 field-on/10 field-off	MF	Double Strand Breaks	[29]
	50 Hz	1 mT	24 h	MF		[30]
	50 Hz	1 mT	15 h	EMF		[31]
	50 Hz	1 mT	15 h, 5′ field-on/10′ field-off	MF	Chromosome aberrations	[29]
	50 Hz	1 mT	2–24 h, 5′ field-on/10′ field-off.	MF	Micronucleus induction	[29]

AC: alternating current;cAMP:adenosine monophosphate; CW: continuous wave;DSBs: double strand breaks; EF: electric fields; EMF: electromagnetic fields; GSH: reduced glutathione; iNOS: inducible nitric oxide synthetase; MF: magnetic field; MW: microwave; rms: root mean square; S: power density, SAR: absorption rate; ROS: reactive oxygen species; SOD: superoxide dismutase.
